# Supplementary material for: Polyketide synthase gene clusters in the ericoid mycorrhizal fungus Oidiodendron maius: genome-wide prediction, expression profiling, and functional characterization of knockout mutants
Source: Front Plant Sci. 2026 Sep 16;17:1924430. doi: 10.3389/fpls.2026.1924430 (PMC13623903; doi:10.3389/fpls.2026.1924430)
Supplement: Supplementary file 1 [file SupplementaryFile1.zip › Supplementary Materials/Supplementary Folder 1.zip!/Oidma1_AssemblyScaffolds_Repeatmasked/knownclusterblast/cluster40/jgi.p_Oidma1_133360_mibig_hits.html]

| MIBiG Protein | Description | MIBiG Cluster | MiBiG Product | % ID | % Coverage | BLAST Score | E-value |
| --- | --- | --- | --- | --- | --- | --- | --- |
| AAK33077.1 | Tri8 | BGC0001278 | Nivalenol\_/\_deoxynivalenol\_/\_3-acetyldeoxynivalenol\_/ | 50 | 96.4200477327 | 366 | 3.3e-101 |
| AAK33086.1 | TRI8 | BGC0001277 | Nivalenol\_/\_deoxynivalenol\_/\_3-acetyldeoxynivalenol\_/ | 50 | 97.1360381862 | 355 | 9.8e-98 |
| AAK53582.1 | TRI8 | BGC0000931 | Trichothecene\_biosynthetic\_gene\_cluster | 50 | 97.1360381862 | 352 | 4.9e-97 |
| AAK53574.1 | TRI8 | BGC0000930 | Trichothecene\_biosynthetic\_gene\_cluster | 48 | 96.4200477327 | 345 | 1e-94 |
